# Carcinoma Skin Reaction

**Published:** 1913-02

**Authors:** 


Carcinoma Skin Reaction.—Drs. Hans Lisser and Arthur Bloomfield (*Bulletin of John Hopkins Hospital,* December, 1912), tabulate 62 cases of verified cancer, 2/3 positive; 94 control cases, 91.6 per cent negative; indicating that conclusions should be drawn rather from positive than from negative reactions. Moss, following Landsteiner, showed that every human being had an apparently lifelong characteristic, with regard to positive or negative agglutination or haemolysis, there being obviously four groups. For the test, red corpuscles from normal individuals of group 4 must be used—neither agglutinated nor haemolysed by any sera in vitro. Fifteen c. c. of blood from the known normal person is withdrawn from a vein of the forearm the day before the test and mixed with sodium citrate solution to prevent clotting. The corpuscles are centrifugalized, washed three times with normal salt solution, made into a 20 per cent. suspension and left over night in the ice chest. One-third to one-half c. c. is injected (never later than 24 hours after preparation) subcutaneously in the patient's forearm. A positive reaction begins about 3 to 5 hours later and reaches its height in 6 to 8 hours. The injection should be made in the morning so as to allow inspection by daylight. A positive reaction consists in an irregular oval, raised spot, brownish red to maroon, with frequently a bluish or purple border. It is slightly boggy to touch and often tender. On subsiding, an ordinary ecchymosis is left. A positive or negative test after a previous positive one and operation, has some prognostic value.

